# Video-Assisted Mini-Open Sublay (VAMOS): A Simple Hybrid Approach for Lateral Incisional Hernias

**DOI:** 10.3389/fsurg.2018.00029

**Published:** 2018-04-04

**Authors:** Robert Schwab, Joachim Sahm, Arnulf Gregor Willms

**Affiliations:** Department of General, Visceral and Thoracic Surgery, German Armed Forces Central Hospital, Koblenz, Germany

**Keywords:** lateral incisional hernia, hybrid technique, sublay, mesh repair, minimally invasive surgery

## Abstract

**Purpose:**

The purpose is to present a new hybrid approach of lateral incisional hernia repair associated with reduced operative trauma and anatomically optimal mesh placement.

**Methods:**

Video-Assisted Mini-Open Sublay (VAMOS) consists of a laparoscopic atraumatic dissection of the hernia sac, diaphanoscopy, laparoscopically-assisted closure of the fascial gap and mesh placement in sublay position through a minimized skin incision. Feasibility of this concept was assessed in a cohort of 7 consecutive patients.

**Results:**

VAMOS approach was feasible in all 7 patients. Median hernia size was 8 cm, the median skin incision width was 7.7 cm. Median operative time was 86 min. In all patients a sufficient mesh overlap on all sides of the fascial gap was ensured. On short-term follow-up no procedure related complications were recorded, seroma formation occurred in 2 patients. Pain medication was necessary for median 4.9 days. There was no need for pain medication on day 14, whatsoever.

**Conclusion:**

Initial VAMOS results show that the technique is simple, time-saving and safe. It provides a substantial reduction in postoperative pain compared to an open approach. Through implantation in the intermuscular sublay position and minor access-related trauma, it is possible to achieve a biomechanically optimal mesh position, to lay the foundations for adequate remodelling of the abdominal wall, and to prevent recurrence as well as local complications. All in all, VAMOS appears to have several advantages over current surgical strategies.

## Introduction

The surgical care of lateral incisional hernias is particularly challenging (1, 2). These hernias occur after open urological surgery of the kidney or efferent urinary system, but also after visceral surgery via a lateral approach such as conventional appendectomies, hemicolectomies or laparoscopically assisted sigmoidectomies with transverse extraction incision (3). Because of the relatively low incidence rate, only a small number of publications are available and there are no standardised evidence-based surgical methods (2, 4, 5). The literature is full of case reports or case series with a small number of cases which present different strategies with different results (1, 5).

Nevertheless, the management of lateral abdominal wall hernias is of major economic and individual medical relevance. Lateral abdominal wall hernias develop complications such as incarcerations more often than midline hernias (6–8).

Due to the particular anatomy of lateral hernias, it is not possible to completely transfer the techniques described for midline hernias (3). Laterally, there are more anatomical, but much thinner muscle and tissue layers, but also much less soft tissue than medianly. The management of lateral hernias is complicated by the mostly simultaneous relaxation of lateral muscles due to nerve lesions in primary surgery which cause a marked bulging of the lateral abdominal wall. This is a significant cause of the complaints and lower quality of life of this patient group (9).

On the whole, the development of new hernia surgery techniques has gathered great momentum over the recent decade and even more so in recent years (10). While it is true that the Rives–Stoppa technique in sublay hernia repair has the advantage of a biomechanically optimal position, it is also associated with relevant procedure-related morbidity on account of major access-related trauma (11, 12). In addition to pain, nerve damage and superficial wound infections, adverse effects also include deep and major mesh infections (11, 12). The IPOM technique (intra peritoneal onlay mesh) was developed to minimise access-related trauma especially if it is performed laparascopically. However this technique entails the inherent risk of relevant major complications such as arrosion of hollow organs with subsequent perforation and peritonitis resulting from the intraperitoneal mesh position (13, 14). This has also been shown in recent publications and discussions at recent hernia congresses (14). The ideal mesh position for the repair of lateral incisional hernias is thus intermuscular and extraperitoneal; placement between the internal and the external oblique muscles is recommended (2). This helps prevent damage to neurovascular bundles (5, 9). In addition, laparoscopic access is preferable in lateral hernias because intestinal components that are often to be found in the hernia sac can otherwise only be safely managed through a large open incision.

A possible solution is a technique that uses the synergistic effects of both surgical methods: *minimally invasive* surgery combined with a mesh positioned *between* the muscle layers.

In the following, we present a new hybrid technique for managing lateral hernias: video-assisted mini-open sublay (VAMOS).

## Material and Methods

### Patients

All patients treated according to the VAMOS procedure during the year 2017 were enrolled. Feasibility of this concept was assessed in a cohort of 7 consecutive patients with a lateral abdominal wall incisional hernia

## Surgical Technique

### Patient Position

Following intubation and general anaesthesia, the patient is placed in a 30-degree recovery position on a vacuum mattress. Both the patient's upper body and legs are lowered, with the hernia at the hinge point. The arm contralateral to the hernia is placed at a right angle, the arm ipsilateral to the hernia is placed onto a surgical frame or Guedel holder. The patient's legs and ankles are padded as needed. The monitor of the laparoscopic unit is positioned behind the patient. As the inclination angle along the longitudinal axis can be changed, this position provides favourable surgical conditions for both the laparoscopic and the open part of the intervention.

## Surgical Steps

Step 1: Infraumbilical mini-laparotomy and insertion of an 11 mm optical trocar. Insufflation of abdomen with CO2 to a maximum of 15 mmHg to produce a pneumoperitoneum. Diagnostic laparoscopy and in particular inspection of the hernia sac and its contents to check the feasibility of the next steps.

**Figure 1:**
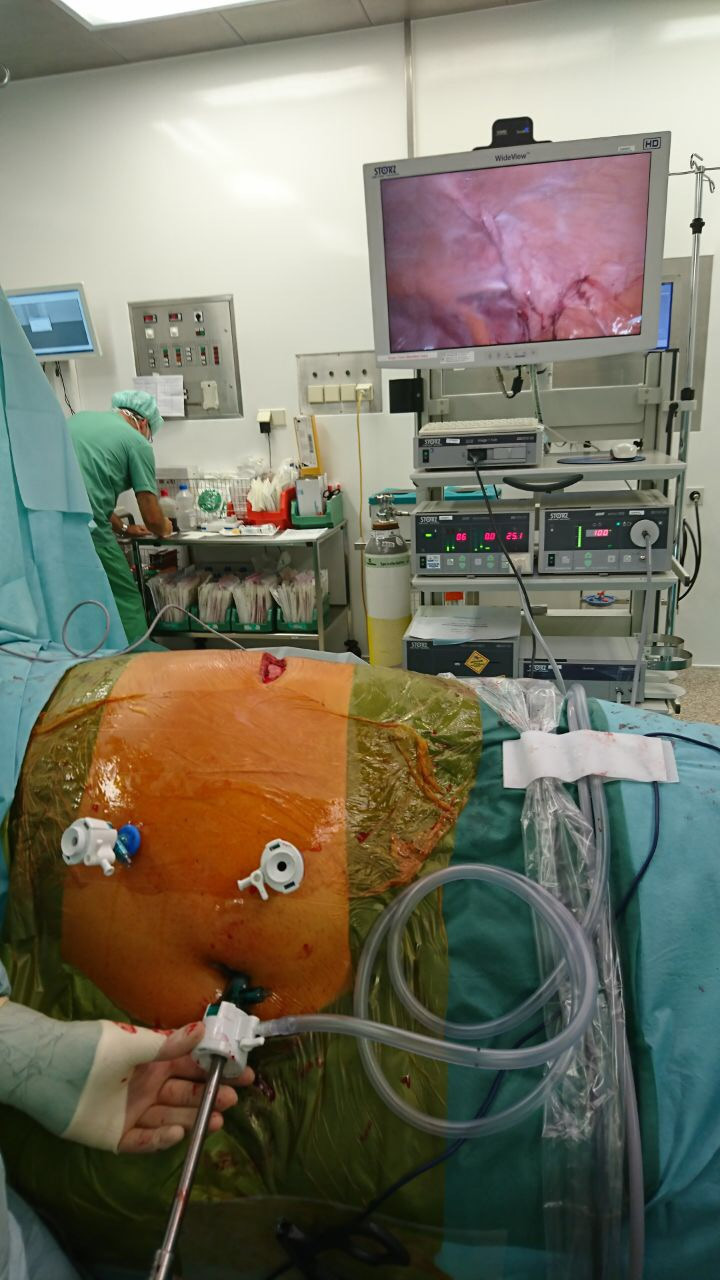
Operative setting. Trocars for instruments are inserted cranially and caudally in an appropriate angle to the hernia (note the skin incision on the left flank); whereas the optic trocar is placed infraumbilically.

Step 2: Insertion of two 5 mm working trocars cranially and caudally in a suitable angle to reach the hernia and perform a proper diagnostic laparoscopy (Figure 1).

**Figure 2:**
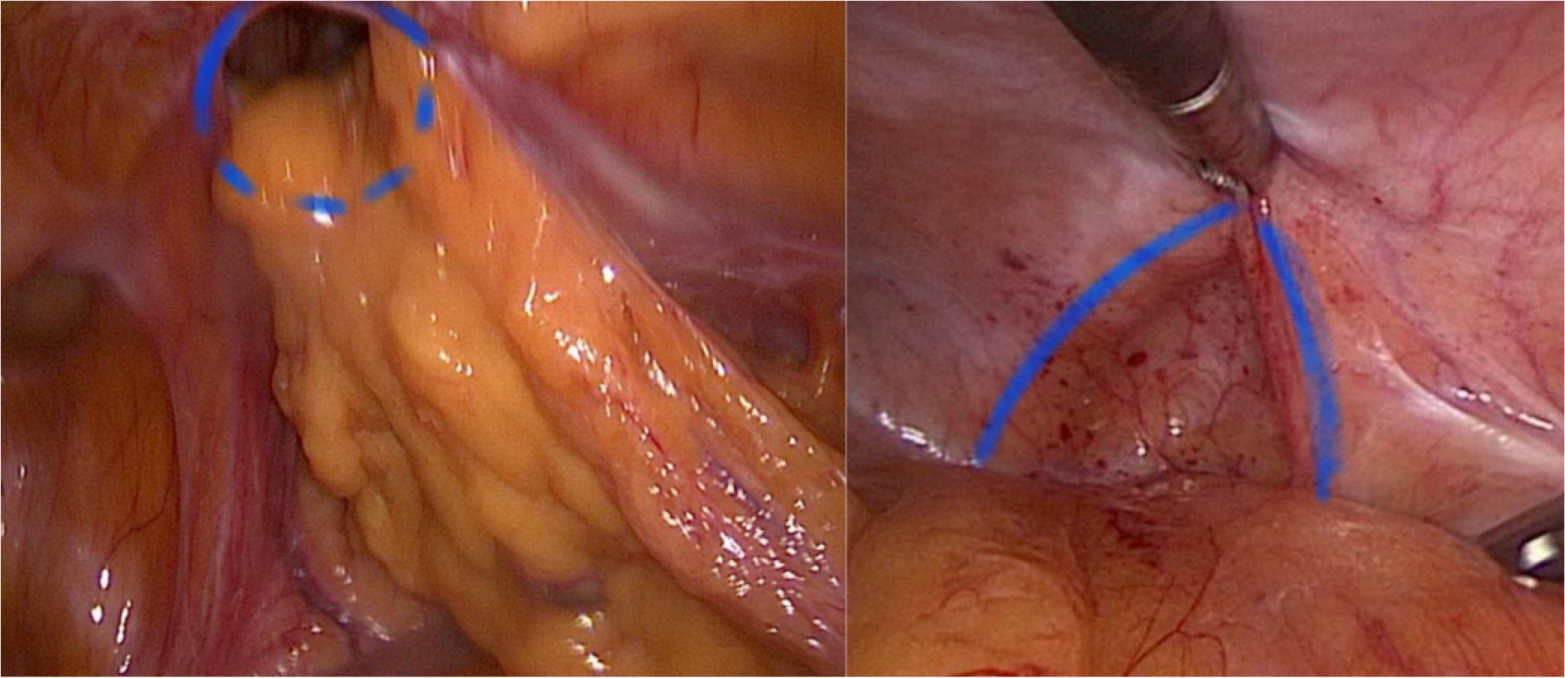
Fascial edges are marked with blue lines. The left part of the image depicts herniated parts of the omentum.

Step 3: Insertion of endoscopic instrument. We recommend atraumatic grasping forceps and bipolar endoscopic scissors or even better a 5 mm Maryland LigaSure Dissector (Covidien/Medtronic, Minneapolis, USA). Atraumatic removal of hernia sac contents from hernia sac and successive repositioning in the intraperitoneal direction (Figure 2). Depending on the hernia position, it may also be necessary to reposition the greater omentum, the small intestine, and colon parts. Separation of the ascending/descending colon or sigmoid colon from retroperitoneal adhesions along Toldt's fascia analogous to laparoscopic colon surgery.

**Figure 3:**
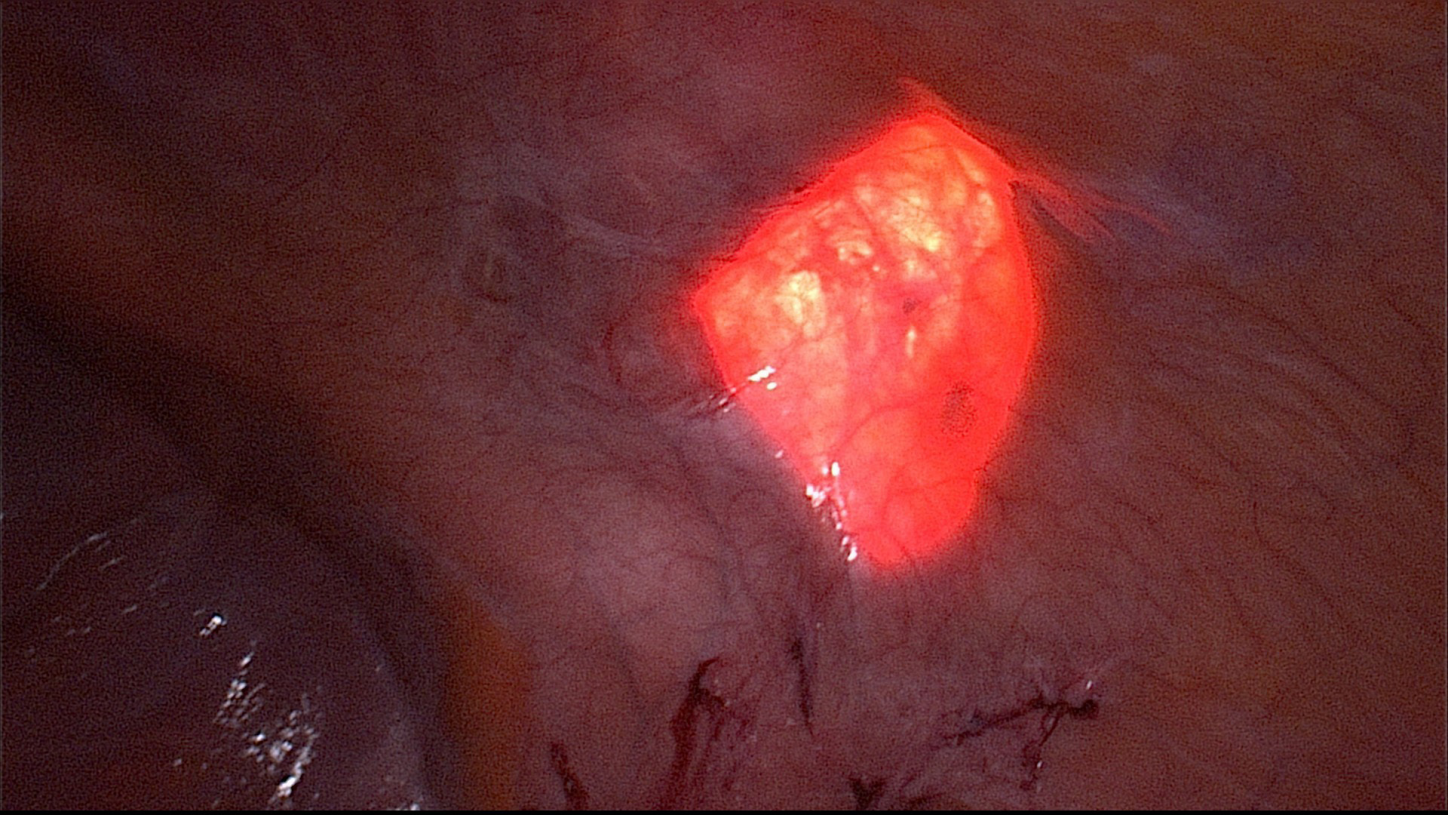
Diaphanoscopy of the fascial gap from intraabdominal perspective. The hernia margins are clearly visualized by the translucency of the fascial gap.

Step 4: Diaphanoscopy and measuring of the hernia defect (Figure 3). Incision exactly above the defect. For this purpose, longitudinal rotation of the surgical table until the hernia defect points exactly upwards. Diaphanoscopically or laparoscopically controlled exposure of the hernia defect without opening the peritoneum; repositioning of the hernia sac.

**Figure 4:**
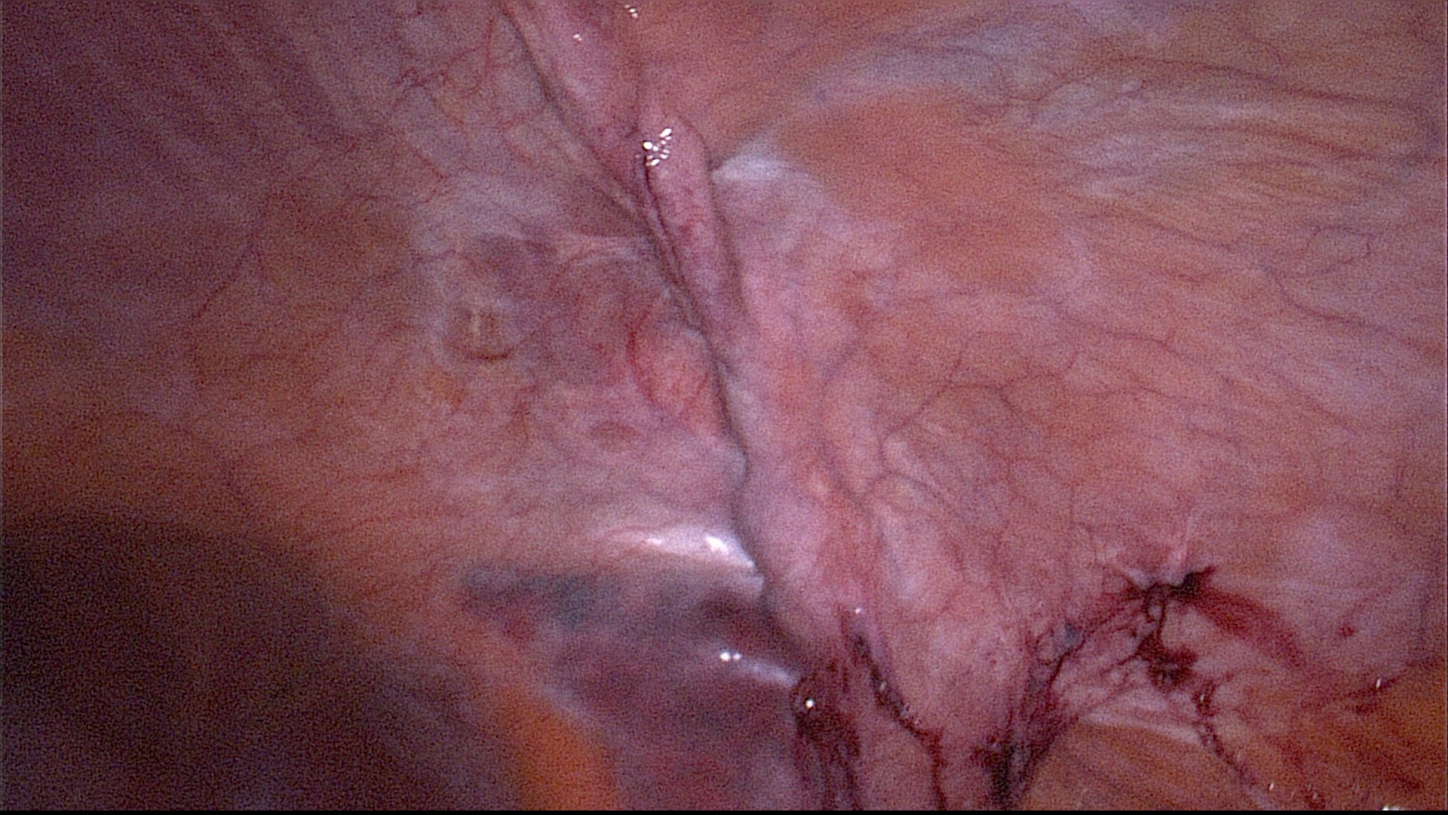
Intraabdominal view on the former hernia. The fascial gap is has been closed with a running suture.

Step 5: Continuous overcast closure of the hernia defect in the transverse and internal oblique abdominal muscle under laparoscopic control with an external non-absorbable suture (Figure 4). This can also be done laparoscopically with barbed suture or internal running suture with the same result.

To this end, insertion of Roux retractors and shifting the skin incision over the previously exposed hernia defect.

Step 6: Preparation of an adequate mesh site to ensure overlapping of at least 3 cm on all sides. Insertion of Roux abdominal retractors and connection of the fascia of the external oblique muscle by a Mikulicz clamp and retraction. This is how an adequate mesh site is prepared atraumatically using blunt and sharp instruments. Special attention to and protection of neurovascular bundles is mandatory to avoid further relaxation and perfusion problems. A low-pressure pneumoperitoneum (8 mmHg) has proved successful for this step and the subsequent mesh implantation. This produces an elastic counterpressure of the lower layer, which greatly simplifies preparation. Large-scale preparation is cranial up to the costal arch, caudal up to the iliac crest, dorsal up to the lumbar muscles, and ventral up to the linea semilunaris.

**Figure 5:**
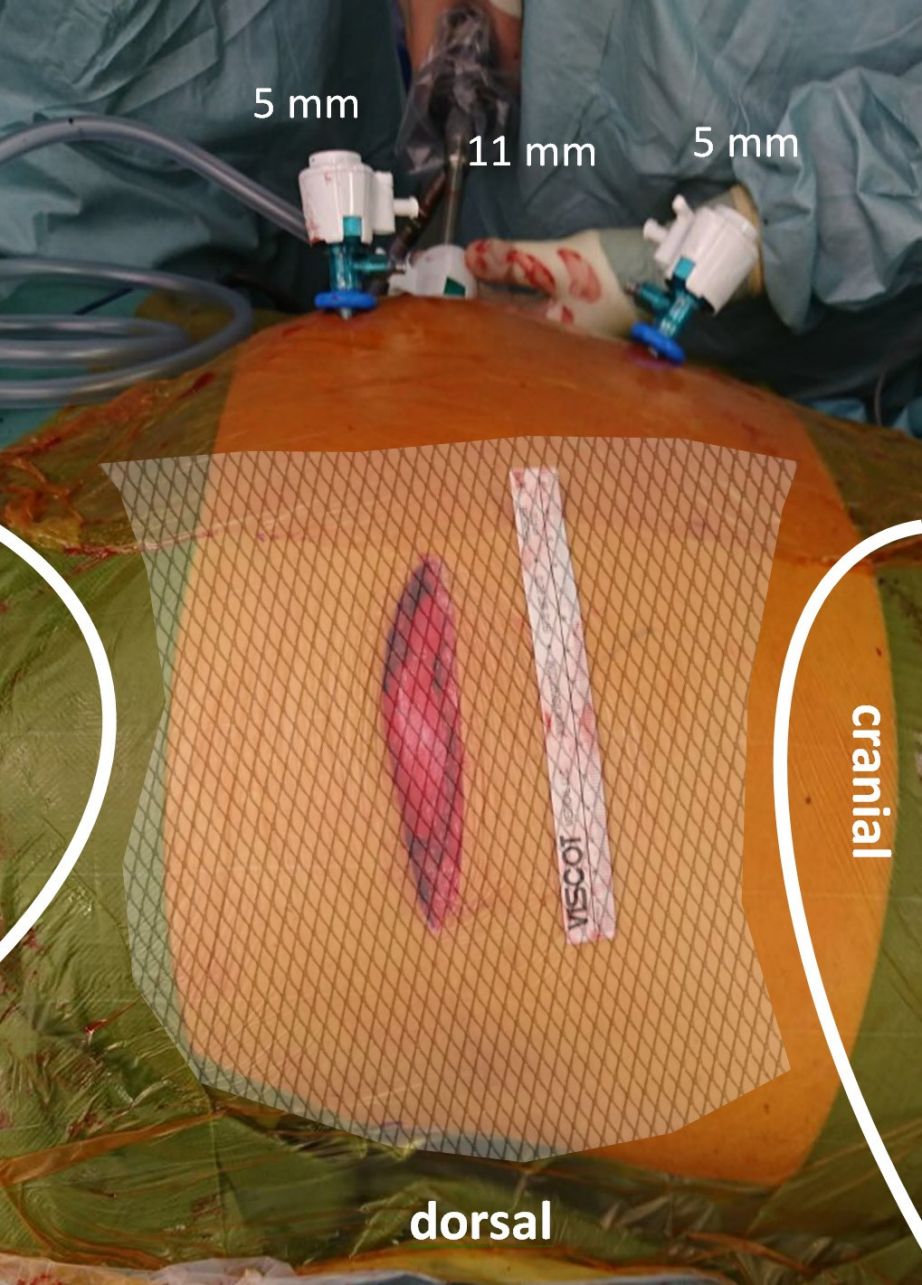
“Mini-open” skin incision before wound closure. The mesh has been placed in sublay position with a sufficient overlap in all directions.

Step 7: Measurement of the mesh site to determine the required mesh size. Mesh implantation via the mini-open approach from outside and stretching of the mesh evenly over the site up to the predetermined anatomical limits (Figure 5). We always use DynaMesh®-Cicat transverse meshes (Dahlhausen, Germany) of different sizes to ensure a sufficient overlap of the fascial gap (Table 1). Mesh fixation with 2–4 mL fibrin glue.

**Table 1:** Characteristics of study population.

n	7
Sex	M 2 (30.0%)F 5 (70.0%)
Age in years	77 (56–81)
BMI in kg/m²	28.2 (25.9–30.0)
ASA status	II 3 (42.9%)III 4 (57.1%)
Preoperative pain (NRS)	4 (2–6)
Hernia diameter in cm	8.0 (4–10.6)
Hernia area in cm²	40.0 (16–61.7)
Length of skin incision in cm	7,7 (5.5–10)
Mean mesh size in cm²	19,9 × 14,4 (12 × 12 - 30 × 20)
Duration of surgery in min	86.0 (58.0–159)
Mesh area in cm²	298 (144–600)
Length of stay in days	5.6 (4–8)
Postoperative pain on day 7 (NRS)	1.0 (0–3)
Usage of pain medication in days	4.9 (3–7)
Postoperative pain on day 14 (NRS)	0.8 (CI: 0.4–1.7)
Complications according to Clavien and Dindo	None
Seroma	2 (28.6%)

Figures are given as absolute numbers and percentages, whereas metric variables are shown with median and CI.

N, number of cases; M, male; F, female; BMI, body mass index; ASA, American Society of Anaesthesiologists score; NRS, numerical rating scale (0–10).

Step 8: Removal of endoscopic instruments and re-elevation of the upper body and legs. Closure of the external oblique muscle with a slowly absorbable sling suture. Skin suture. No drain is inserted.

## Patients

During the year 2017 we registered 7 patients with a lateral abdominal wall incisional hernia who were to be treated with VAMOS. Since March 2016, our hospital has been certified as a centre of reference for hernia surgery according to the requirements of the German Society for General and Visceral Surgery (DGAV).

**Figure 6:**
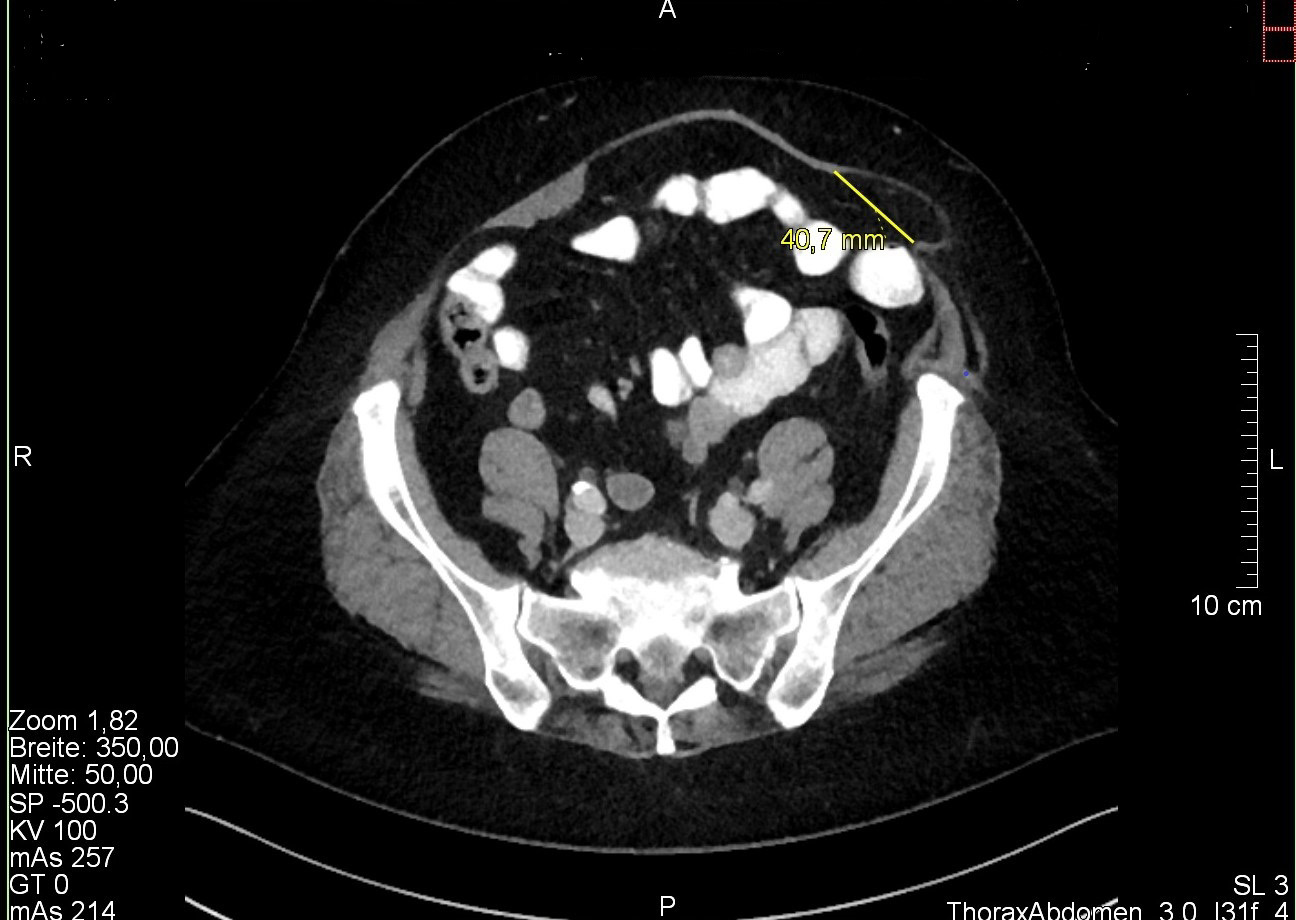
Off-midline hernia with a hernia diameter of 4 cm.

During the treatment we documented biometric data, hernia-specific characteristics, surgery-specific data such as duration of surgery and the size of mesh implanted, as well as the results of post-inpatient follow-up. For hernia diagnosis, all patients had a preoperative ultrasound examination using a 7.5 MHz linear transducer to measure hernia dimensions. The maximum diameter and total area (area = (length*width)/2) were determined. Furthermore Figure 6 shows a CT-scan of one of the treated off-midline hernias with a hernia diameter of 4 cm (Figure 6).

All patients received an epidural catheter before surgery. Additionally, they were administered 1 g metamizole as a standard postoperative measure. This amount was modified over time depending on the level of pain. Half an hour prior to skin incision, each patient received 1.5–3 g of cefuroxime intravenously as preoperative, weight-based, single-shot antibiotic prophylaxis. Postoperatively, all patients wore an abdominal bandage until the day of discharge to ensure sufficient compression and to avoid mesh dislocation and seroma.

Following discharge from hospital, all patients had a follow-up examination that included a standardised questionnaire, a physical examination, blood tests, as well as standardised sonography using a 7.5 MHz linear transducer.

## Results

Data collection details are shown in Table 1.

## Hernia Characteristics

Five patients had hernias after open urological operations in the lumbar region. One patient had a combined hernia following appendectomy and a parallel ipsilateral inguinal hernia and one patient had a trocar hernia in the left lower abdomen. Two hernias were located on the right, the other ones on the left.

The average hernia area was 40 cm², the maximum diameter was 8 cm on average. The average preoperative pain intensity level was 4 on the numerical rating scale (NRS).

### Surgery Characteristics and Hospital Stay

All operations were elective interventions and each of them used the VAMOS approach. In particular, laparoscopic surgery succeeded in all cases in completely freeing the hernia sac from adhesions and repositioning it. Overlapping of at least 3 cm on all sides was ensured in all cases. There were no intraoperative complications. On average, the epidural catheter was removed on day 3 (2–5). At discharge, none of the patients needed analgesics.

### Results of Follow-Up Examination

At the follow-up examination, which on average took place on day 11.3 (day 9 to 13), undisturbed wound healing was seen in all patients. There were no postoperative complications in our population. There was no early recurrence. This was ruled out sonographically and clinically. In addition, ultrasonography ruled out mesh dislocation in all cases. In 2 out of 7 patients (28.6%), a seroma border exceeding 1 cm in diameter was detected by ultrasonography. At follow-up no clinically relevant bulging was seen. None of the patients needed analgesics.

## Discussion

There is currently no standard surgical technique for lateral incisional hernias (2, 5). Various techniques have been described in case reports and small case series (1, 5). Despite their relatively low incidence, however, such hernias remain a relevant issue and a major challenge. They are accompanied by a high rate of incarcerations and strangulations, are normally symptomatic, and affect the quality of life of those concerned (5–9).

In addition, a relaxation of lateral abdominal muscles as a result of nerve lesions caused during primary surgery complicates the anatomical stability and functional capacity of the lateral abdominal wall in the hernia region (5). Primary suture closure of the hernia is ruled out because of high recurrence rates so that an implantation of foreign material is obligatory (15). However, the anatomy of the lateral abdominal wall and its hernias differs substantially and fundamentally from the anatomy of midline hernias (2). Mesh implantation using the open sublay technique therefore is a major surgical challenge in lateral hernias (3, 5, 12).

The VAMOS technique presented in this paper for lateral incisional hernias of the abdominal wall is a hybrid technique combining a minimally invasive laparoscopic approach with a mini-open strategy. It is designed to combine the benefits of laparoscopy and thus patient safety with the advantages of the sublay position in lateral incisional hernias of the abdominal wall. The aim is user-friendly, time-saving hernia management with as few complications as possible.

The results presented here are promising. They suggest a substantial advantage of the technique over previous therapeutic alternatives such as IPOM, open sublay, and onlay. IPOM is an alternative technique that has the advantage of laparoscopic preparation und simple and atraumatic mesh implantation, but it is associated with relevant intraabdominal early and late complications (13, 14). It is true that the open lateral sublay does grant an ideal mesh position, but it also entails a relevant risk of wound healing problems including mesh infections and the risk of surgery-related secondary damage (11, 12). The onlay technique cannot be used because it is associated with a biomechanically unfavourable mesh position as well as mesh infections due to less lateral soft-tissue coverage. The VAMOS technique is only a minor challenge for laparoscopically experienced surgeons and does not require the introduction of new equipment or a completely new surgical technique. It relies on available capabilities and resources.

VAMOS is thus the logical consequence of efforts in recent years: extraperitoneal placement of foreign material where possible while simultaneously minimising access-related trauma (10). It grants an ideal, intermuscular and extraperitoneal mesh position. For example, the mesh can be placed between the internal and the external oblique muscles. In this way, the neurovascular bundles are preserved, a sufficient dorsal overlap is ensured, and secondary damage to the neurovascular bundles is avoided. Preserving the neurovascular bundles is all the more important as lateral incisional hernias following access trauma are often associated with relaxation resulting from iatrogenic denervation of lateral abdominal muscles (5). Further circulation and innervation disorders should therefore best be avoided (2). Adequate preparation is also possible in the caudal direction towards the iliac crest, in the cranial direction towards the costal arch, and in the ventral direction. For fixation below the costal arch, the internal oblique muscle must be separated from the lower edge of the rib cage. To achieve an overlap in the ventral direction towards the linea semilunaris, the posterior lamina of the rectus sheath is incised (16).

Osseous fixation with costal or pelvic bone is also possible and is recommended in pre-existing excessive bulging to tighten the lateral abdominal wall and to avoid postoperative bulging (12).

Despite all the constraints associated with our small population, our VAMOS population had much shorter durations of surgery in comparison to studies examining open sublay implantation (3–5, 10, 12). This may be due to clear and comfortable laparoscopic hernia sac preparation. In line with known studies that compare open and minimally invasive approaches, we also expect lower postoperative wound infection rates and pain incidence (17).

Due to the time-consuming preparation involved in an incision through the old scar, Zieren et al. present an approach that uses a median incision and thus ensures easier preparation of the hernia (5). Median incision is, however, another significant trauma and is associated with further risks (wound infections, incisional hernias, etc.). In their publication, Sun et al. introduce the TAPE technique, a lateral, laparoscopic IPOM technique, which produced favourable results in 14 patients (18). The mean duration of surgery was slightly lower than in our population. However, it remains doubtful whether the IPOM technique induces remodelling expected from foreign material implantation in the same way as a mesh in sublay position. In addition, obligatory stapling is a risk for chronic pain syndrome (19). Similarly, postoperative bulging is not prevented by the IPOM technique (12).

Moreover, due to the mesh position, the VAMOS technique does not use the expensive, coated meshes required by IPOM. A mesh for the sublay technique costs only a fraction of the price of an IPOM mesh. The use of a drain is not necessary due to the fact, that only in a few cases seromas were observed, which do not need any further intervention.

We can thus see certain advantages of the VAMOS technique compared with other techniques also examined in small populations. The results presented in this paper are, of course, subject to the constraints of the case series. Further studies and longer follow-up intervals are necessary to determine the value and significance of the VAMOS technique.

## Conclusion

Initial VAMOS results show that the technique is simple, time-saving and safe. It provides a significant reduction in postoperative pain. Through implantation in the intermuscular sublay position and minor access-related trauma, it is possible to achieve a biomechanically optimal mesh position, to lay the foundations for sufficient remodelling of the abdominal wall, and to prevent recurrence as well as local complications. All in all, VAMOS appears to have several advantages over current surgical strategies.

## Ethics Statement

Ethical approval was not required for this study in accordance with the local legislation and institutional requirements. This is confirmed by the regional ethics committee on the basis that the patients received only accepted standard care and no data were recorded, which were only study related.

Informed consent was obtained from all individual participants included in the study. All procedures performed in studies involving human participants were in accordance with the ethical standards of the institutional and/or national research committee and with the 1964 Helsinki declaration and its later amendments or comparable ethical standards. This article does not contain any studies with animals performed by any of the authors.

## Author Contributions

RS, JS and AW developed the described technique, AW and RS set up the study design, JS and AW collected the data prospectively, AW performed the data analysis, AW and RS wrote the manuscript

## Conflict of Interest Statement

The authors declare that the research was conducted in the absence of any commercial or financial relationships that could be construed as a potential conflict of interest.

## References

[1] Pulikkottil BJ Pezeshk RA Daniali LN Bailey SH Mapula S Hoxworth RE Lateral abdominal wall defects: the importance of anatomy and technique for a successful repair. *Plast Reconstr Surg Glob Open* (2015) 3(8):e481 10.1097/GOX.0000000000000439 26495194 PMC4560214

[2] Stumpf M Conze J Prescher A Junge K Krones CJ Klinge U The lateral incisional hernia: anatomical considerations for a standardized retromuscular sublay repair. *Hernia* (2009) 13(3):293–7. 10.1007/s10029-009-0479-0 19214648

[3] Patel PP Warren JA Mansour R Cobb WS Carbonell AM A large single-center experience of open lateral abdominal wall hernia repairs. *Am Surg* (2016) 82(7):608–12. 27457859

[4] Edwards C Geiger T Bartow K Ramaswamy A Fearing N Thaler K Laparoscopic transperitoneal repair of flank hernias: a retrospective review of 27 patients. *Surg Endosc* (2009) 23(12):2692–6. 10.1007/s00464-009-0477-4 19462203

[5] Zieren J Menenakos C Taymoorian K Müller JM Flank hernia and bulging after open nephrectomy: mesh repair by flank or median approach? Report of a novel technique. *Int Urol Nephrol* (2007) 39(4):989–93. 10.1007/s11255-007-9186-x 17333509

[6] Esposito TJ Fedorak I Traumatic lumbar hernia: case report and literature review. *J Trauma* (1994) 37(1):123–6. 8028048

[7] Heniford BT Iannitti DA Gagner M Laparoscopic inferior and superior lumbar hernia repair. *Arch Surg* (1997) 132(10):1141–4. 10.1001/archsurg.1997.01430340095017 9336516

[8] Stamatiou D Skandalakis JE Skandalakis LJ Mirilas P Lumbar hernia: surgical anatomy, embryology, and technique of repair. *Am Surg* (2009) 75(3):202–7. 19350853

[9] Chatterjee S Nam R Fleshner N Klotz L Permanent flank bulge is a consequence of flank incision for radical nephrectomy in one half of patients. *Urol Oncol* (2004) 22(1):36–9. 10.1016/S1078-1439(03)00099-1 14969802

[10] Schwarz J Reinpold W Bittner R Endoscopic mini/less open sublay technique (EMILOS)-a new technique for ventral hernia repair. *Langenbecks Arch Surg* (2017) 402(1):173–80. 10.1007/s00423-016-1522-0 27766419

[11] Petro CC Posielski NM Raigani S Criss CN Orenstein SB Novitsky YW Risk factors for wound morbidity after open retromuscular (sublay) hernia repair. *Surgery* (2015) 158(6):1658–68. 10.1016/j.surg.2015.05.003 26100569

[12] Phillips MS Krpata DM Blatnik JA Rosen MJ Retromuscular preperitoneal repair of flank hernias. *J Gastrointest Surg* (2012) 16(8):1548–53. 10.1007/s11605-012-1890-x 22528575

[13] Schaaf S Richardsen I Schwab R Güsgen C Willms A [Unusual cause of mechanical small bowel obstruction following incisional hernia repair]. *Chirurg* (2016) 87(10):881–3. 10.1007/s00104-016-0216-z 27316709

[14] Yang GPC From intraperitoneal onlay mesh repair to preperitoneal onlay mesh repair. *Asian J Endosc Surg* (2017) 10(2):119–27. 10.1111/ases.12388 28547932

[15] Conze J Klinge U Schumpelick V Narbenhernien. *Chirurg* (2005) 76(9):897–910. 10.1007/s00104-005-1072-4 16133556

[16] Schumpelick V Klinge U Junge K Stumpf M Incisional abdominal hernia: the open mesh repair. *Langenbecks Arch Surg* (2004) 389(1):1–5. 10.1007/s00423-003-0352-z 14745557

[17] Kaafarani HM Kaufman D Reda D Itani KM Predictors of surgical site infection in laparoscopic and open ventral incisional herniorrhaphy. *J Surg Res* (2010) 163(2):229–34. 10.1016/j.jss.2010.03.019 20605590

[18] Sun J Chen X Li J Zhang Y Dong F Zheng M Implementation of the trans-abdominal partial extra-peritoneal (TAPE) technique in laparoscopic lumbar hernia repair. *BMC Surg* (2015) 15:118 10.1186/s12893-015-0104-3 26507827 PMC4624658

[19] Schwab R Schumacher O Junge K Binnebösel M Klinge U Becker HP Biomechanical analyses of mesh fixation in TAPP and TEP hernia repair. *Surg Endosc* (2008) 22(3):731–8. 10.1007/s00464-007-9476-5 17623239

